# Metabolic Health: The Interplay Between NAFLD, MAFLD, MASLD, and Cardiovascular Disease

**DOI:** 10.3390/life16071197

**Published:** 2026-07-20

**Authors:** Monika Różycka-Kosmalska, Marcin Kosmalski

**Affiliations:** 1 Department of Electrocardiology, Medical University of Lodz, 92-213 Lodz, Poland; monika.rozycka-kosmalska@umed.lodz.pl; 2 Department of Clinical Pharmacology, Medical University of Lodz, 90-153 Lodz, Poland

## 1. Recent Developments, Knowledge Gaps, and How This Special Issue Addresses Them

### 1.1. Recent Developments in the Field

The last decade has transformed the conceptual landscape of fatty liver disease in three ways. First, nomenclature and diagnostic criteria have evolved from the historical “non-alcoholic fatty liver disease” framework to the metabolically anchored “metabolic dysfunction-associated fatty liver disease” and, most recently, to the 2023 AASLD–EASL consensus term “metabolic dysfunction-associated steatotic liver disease”—a change that tightens the link between steatosis and cardiometabolic risk [1]. Second, the therapeutic landscape has shifted decisively: in addition to established agents acting on cardiometabolic risk factors (statins, glucagon-like peptide-1 receptor agonist (GLP-1 Ras), sodium–glucose cotransporter 2 inhibitors (SGLT-2 inhibitors), thiazolidinediones), resmetirom recently became the first pharmacological agent approved by the U.S. Food and Drug Administration (FDA) for metabolic dysfunction-associated steatohepatitis (MASH) and associated liver fibrosis—in a phase 3 trial (n = 966) achieving 25.9–29.9% MASH resolution without worsening fibrosis, compared with 9.7% on placebo [1]. Third, the field has accumulated large-scale epidemiological evidence placing metabolic dysfunction-associated steatotic liver disease (MASLD) as a systemic disease whose principal mortality driver lies outside the liver—cardiovascular diseases, including atrial fibrillation (AF) and heart failure (HF), now constitute the leading cause of death in patients with confirmed MASLD [1].

Methodological innovations have followed. Pooled meta-analyses now routinely aggregate data across millions of subjects: one such analysis combined 16 retrospective cohort studies—totaling approximately 19.5 million individuals followed for a median of 7.2 years—and demonstrated a significant association between MASLD and incident AF (random-effects HR 1.20), unchanged after adjustment for age, sex, body mass index (BMI), systemic arterial hypertension (HA), and type 2 diabetes mellitus (T2DM) [1]. Complementary cross-sectional work, aggregating more than 360,000 subjects across nine studies, has shown that non-alcoholic fatty liver disease (NAFLD) approximately doubles the odds of prevalent AF (OR ~2.07) [1]. In parallel, the development of non-invasive screening indices has enabled population-scale screening without recourse to liver biopsy, while genetic and immunological biomarkers IL-17A/F and TLR4 polymorphisms [2] and homocysteine (Hcy) [3] are emerging as candidate stratification tools. Semaglutide has emerged as the prototypical example of a therapy reducing AF incidence by 42% in trials encompassing 12,651 high-risk individuals followed for approximately 68 months—the largest reported effect on a cardiovascular endpoint attributable to a metabolic drug class in this population [1].

### 1.2. Residual Knowledge Gaps

Despite these advances, the Special Issue’s editorial framework identifies several persisting gaps as genuinely unresolved. First, although the association between MASLD and cardiovascular disease (CVD) is firmly established, a shortage of data remains on which modifiable risk factors are most relevant in subgroups of MASLD patients, on the precise pathomechanisms that link the two disease domains, and on therapeutic regimens that would simultaneously reduce hepatic steatosis and prevent cardiovascular events [1]. Second, the choice of optimal diagnostic tool is unsettled: liver biopsy remains the gold standard but is invasive and resource-intensive; ultrasound and imaging modalities show variable accuracy; non-invasive serum biomarkers remain population-dependent and have been validated primarily in selected settings [3,4]. Third, the natural history across the full life course is incompletely mapped—pediatric MASLD, adolescent sarcopenia-related NAFLD, working-age adults, and elderly patients with cardio-renal phenotypes each present distinct clinical pictures, but harmonized longitudinal data linking all four are scarce [3,5,6]. Fourth, mechanism-targeted interventions remain largely hypothetical: while the Th2-RAS axis [7], the IL-17–TLR4 innate-immunity pathway [2], and adipose-tissue macrophage polarization have been mechanistically implicated in MASLD, no pharmacological agent specifically targets these loci. Fifth, drug-specific long-term cardiovascular outcomes are unknown for the new generation of MASLD drugs (including resmetirom and forthcoming GLP-1/SGLT-2 combinations). Sixth, the distinction between population-specific effect sizes and universal mechanisms remains methodologically underappreciated, as demonstrated by the geographical and demographic heterogeneity of biomarker performance [3,5,8]. Seventh, the relative contribution of sarcopenia, body-composition phenotypes, and lifestyle factors to MASLD severity and progression is incompletely quantified across age strata [6,8].

### 1.3. How This Special Issue Maps onto the Gaps

Each of the nine contributions in this Special Issue was designed to address one or more of these gaps, and collectively they form a complementary response. Table 1 summarizes the explicit mapping.

A non-obvious but important common thread is that every contribution, regardless of its primary axis, touches on cardiovascular risk—whether directly [1,5,7,8], indirectly via cardiometabolic biomarkers [3,6,9], or mechanistically through immunological pathways that contribute to inflammation-driven atherosclerosis [2]. The collection thus concretizes the editorial thesis that MASLD management cannot be confined to hepatology.

### 1.4. Future Research Directions Highlighted by the Special Issue

The contributions collectively delineate five priority research directions. (i) Large, multicenter, prospective validation of non-invasive diagnostic algorithms across populations differing in ethnicity, age, and comorbidity profile, with explicit attention to the elderly, where existing tools such as Fibrosis-4 Index (FIB-4) may lose predictive value [3,4,5]. (ii) Randomized intervention trials addressing MASLD and cardiovascular endpoints simultaneously, particularly comparing metabolic therapies (GLP-1 RAs, SGLT-2 inhibitors, resmetirom) head-to-head and in combination—including long-term follow-up beyond the 5–10-year window captured in current trials [1]. (iii) Mechanistic dissection of immunological endotypes of MASLD beyond the prototypical Th17/TLR4 and Th2/RAS axes, using single-cell liver-tissue profiling in biopsy series correlated with cardiometabolic phenotypes [2,7]. (iv) Pediatric and adolescent natural-history studies that integrate sarcopenia phenotyping, homocysteine monitoring, and resistance-training interventions in longitudinal designs with cardiometabolic endpoints [3,6]. (v) Translational research on natural antioxidant compounds such as white tea, with a need to move from current rodent models to future randomized human trials powered for metabolic and cardiovascular outcomes [9]. The dissemination of these priorities through the present Special Issue is intended to stimulate follow-up work and to accelerate convergence between the currently fragmented research streams that the collection brings together.

## 2. Framing the Problem—Why a Multidisciplinary Editorial Framework Matters

The collection is unified by recognizing NAFLD, metabolic dysfunction-associated fatty liver disease (MAFLD), and MASLD as a single family of related—but diagnostically distinct—entities that collectively constitute one of the most common liver diseases worldwide, with significant cardiovascular, metabolic, renal, and oncologic consequences beyond the liver. The central controversy that animates the entire Special Issue is that although the epidemiological relationship between MASLD and cardiovascular disease has been convincingly demonstrated, there is a shortage of data on the modifiable risk factors for CVD in patients with MASLD, on the pathomechanisms that link the two disease domains, and on the therapeutic regimens that would simultaneously reduce hepatic steatosis and prevent cardiovascular events. The Special Issue is structured around this gap, contributing evidence on each dimension from different populations and methodological perspectives.

A complementary clinical framing places non-pharmacological interventions—Mediterranean diet, regular physical activity, and sustained weight loss—as the cornerstone of post-diagnosis management, while acknowledging the persistent shortage of registered drugs targeting NAFLD directly and the limitations of pharmacological management strategies that still rely to a large extent on findings from small, single-center cohort studies [2,3]. This dual stance—acknowledging the central role of lifestyle intervention while documenting an ongoing pharmacological gap—shapes the structure of the entire Special Issue.

## 3. The Global Burden of MASLD—A Shared Quantitative Anchor

A quantitative anchor unifies the contributions. Morawska and colleagues estimate that approximately 30% of the world’s adult population is affected by MASLD—a prevalence that has risen substantially over the past three decades, in parallel with trends in obesity and T2DM [1]. About 75% of individuals with excess body weight develop MASLD [1]. Once the diagnosis is established, cardiovascular diseases—including AF and HF—constitute the leading cause of mortality, followed by extrahepatic malignancies and complications arising directly from hepatic dysfunction [1].

This reframing of MASLD as a disease whose principal mortality driver lies outside the liver—making cardiologists and internists as central to its management as hepatologists—provides the structural logic for the Special Issue’s organization, beginning with the most consequential CVD association and moving through diagnostic, epidemiologic, mechanistic, and therapeutic dimensions.

## 4. The Cardiovascular Axis—MASLD and Atrial Fibrillation

### 4.1. Mechanistic Crosstalk

Morawska and colleagues provide the most comprehensive mechanistic synthesis in the Special Issue [1]. AF is presented as the most common age-related cardiac arrhythmia, with an estimated 60 million affected individuals worldwide and a lifetime risk of approximately 33% [1]. The review surfaces a striking quantitative fact: in analyses of nine cross-sectional and longitudinal studies encompassing more than 360,000 subjects, NAFLD approximately doubled the odds of prevalent AF (OR ~2.07), while a meta-analysis of 16 retrospective cohort studies aggregating data on approximately 19.5 million individuals followed for a median of 7.2 years found that MASLD is significantly associated with incident AF (random-effects HR 1.20)—a correlation that remained significant after adjustment for age, sex, BMI, HA, and T2DM [1]. Adjustments for adiposity, T2DM, and HA attenuate the association but do not abolish it, and fibrosis severity yields higher risks in some cohorts (e.g., cirrhosis), while ablation populations show stronger effects for arrhythmia recurrence [1]. In a prospective multicenter cohort study of patients with non-valvular AF, NAFLD was diagnosed in 732 of 1735 (42.2%) participants, illustrating the high overlap between the two conditions in clinical practice [1].

At the mechanistic level, the review maps the pathways by which MASLD promotes AF: lipid accumulation in hepatocytes intensifies mitochondrial β-oxidation and cytochrome P450 activity, leading to overproduction of reactive oxygen species that overwhelm antioxidant defenses—superoxide dismutase, catalase, and glutathione peroxidase [1]. The resulting systemic metabolic dysregulation, chronic low-grade inflammation, and release of profibrotic mediators can reach the myocardium, promote atrial fibrosis, and drive electrical remodeling—all core components of atrial cardiomyopathy [1]. Importantly, the genetic architecture of AF (PITX2, KCNN3, TTN, MYH6) does not substantially overlap with variants linked to hepatic lipid metabolism (PNPLA3, TM6SF2), indicating that MASLD elevates AF risk through systemic metabolic and inflammatory pathways rather than shared heredity [1]. Among patients diagnosed with MASLD, T2DM is the most frequent comorbidity—affecting up to 75%—and markedly amplifies the risk of cardiovascular events, malignancy, and liver-related complications [1].

Therapeutically, the review highlights that metabolic and anti-inflammatory interventions may simultaneously address MASLD and AF. In a meta-analysis of 10 randomized clinical trials encompassing 12,651 participants with a median follow-up of approximately 68 months, semaglutide reduced AF incidence by 42% compared with placebo among individuals at high cardiovascular risk—an effect consistent across drug route (oral or subcutaneous), presence or absence of T2DM, and across BMI categories [1]. Resmetirom—a selective thyroid hormone receptor-β agonist—recently became the first pharmacological agent approved by the FDA for MASH and associated liver fibrosis; in a phase 3 trial (n = 966), 25.9–29.9% of patients receiving the drug achieved MASH resolution without worsening of fibrosis, compared with 9.7% on placebo [1]. In a retrospective cohort of 1238 MASLD patients, statin therapy was associated with reduced risk of progression to high-risk fibrosis (HR 0.60 for moderate exposure and 0.61 for high exposure) [1]. The review emphasizes that appropriate anticoagulation remains essential for AF management itself, alongside any metabolic therapy targeting MASLD [1].

### 4.2. Clinical Phenotypes Across AF Subtypes

The mechanistic review is complemented by a prospective single-center observational study by Różycka-Kosmalska, Luzak, and Kosmalski, which—to the best of available evidence—represents the first systematic analysis of clinically inferred MASLD across paroxysmal, persistent, and permanent atrial fibrillation subtypes, integrating cardiometabolic and cardiorenal parameters [5]. The authors enrolled patients hospitalized due to AF in a cardiological department (controls from an outpatient cardiology clinic), with case–control matching on age and sex [5]. A clear phenotypic gradient was observed: MASLD prevalence was lowest in permanent atrial fibrillation (PermAF) (27.1%), compared with paroxysmal atrial fibrillation (PAF) (47.1%), persistent atrial fibrillation (PeAF) (51.4%), and the controls (43.7%) (*p* = 0.0021) [5].

Patients with PermAF exhibited the most advanced comorbidity profile—the highest chronic HF burden (78.6%), the highest chronic kidney disease (CKD) prevalence (71.4%), the highest proportion of heart failure with preserved ejection fraction (HFpEF, 48.6%), the highest FIB-4 score (median 2.67), the lowest triglycerides-to-HDL ratio (TG/HDL) (1.93 vs. 3.32 in other groups; *p* < 0.001), and progressive renal impairment [5]. All four groups were older than the control group (median ages of approximately 60, 65, 70, and 79.5 years in control, PAF, PeAF, and PermAF, respectively; the maximum standardized mean difference reached 1.40), consistent with epidemiological data showing that AF prevalence rises sharply with age and reaches approximately 21.9% in patients over 85 years of age in primary-care cohorts [5]. Across HF phenotypes, heart failure with reduced ejection fraction (HFrEF) was least frequent in PermAF (8.6%), while HFpEF predominated (48.6%, *p* < 0.001) and heart failure with mid-range ejection fraction (HFmrEF) reached its peak prevalence (21.4%, *p* = 0.001) in this group [5].

The authors explicitly caution that the elevated FIB-4 observed in PermAF must be interpreted carefully: this group was substantially older and carried the highest burden of HF and CKD; therefore, FIB-4 most plausibly reflects age and cardio-renal comorbidity rather than histologically confirmed hepatic fibrosis [5]. After matching, MASLD was not an independent predictor of AF presence (OR = 0.96; 95% CI: 0.59–1.46) or of its clinical severity [5]. The data support a model in which early AF is characterized by a metabolic phenotype (higher MASLD prevalence and elevated triglycerides), whereas advanced AF shifts toward a cardio-renal phenotype with progressive deterioration of renal function and, in the most advanced stage, transitions further into a fibrotic, organ-dysfunction pattern, with age as the dominant driver of this progression [5]. This chronological evolution of AF carries important implications for clinicians: risk stratification must account for age and comorbidity burden, and biomarkers validated in younger, metabolically driven cohorts may not be directly applicable to elderly populations with cardio-renal phenotypes.

### 4.3. HA and the Renin–Angiotensin System—An Immunological Bridge

The cardiovascular axis of MASLD extends beyond AF to include HA, addressed by Méndez-García and colleagues through an innovative immunological lens [7]. In an exploratory investigation, the authors assessed the expression of cytokines and key molecules of the renin–angiotensin system pathway in liver tissue from patients with morbid obesity and MASLD, with and without HA [7]. The underlying framework—that the renin–angiotensin system plays a central role in the development and progression of HA, and that chronic inflammation in obesity (linked to liver steatosis) may drive RAS dysregulation—had not previously been characterized at the hepatic level in this patient population [7].

The findings mark a significant advance: a statistically significant correlation was observed between hepatic ACE levels and the Th2 cytokines IL-4, IL-10, and IL-13, with IL-4 and IL-13 emerging as the best predictors of ACE levels in multiple linear regression. Among the cytokines examined, only IL-13 was significantly higher in patients with morbid obesity, MASLD, and SAH than in those without SAH [7]. Mechanistically, the authors propose that the interplay involving ACE and Th2 cytokines may constitute a previously unrecognized hepatic regulatory circuit linking metabolic inflammation to blood pressure regulation [7]. This is the first hepatic-level demonstration of a mechanistic link between Th2-type immunity and the RAS in patients with obesity, MASLD, and SAH, opening a novel avenue for biomarker development and therapeutic targeting [7].

## 5. The Diagnostic Axis—Non-Invasive Tools Across Populations and Ages

### 5.1. The Vietnamese Clinical Experience

This section addresses a key controversy—the absence of an optimal diagnostic tool—through the contribution of Nguyen and Vo, who evaluated the predictive value of four non-invasive indices in a Vietnamese population [4]. The clinical gap is well known: liver biopsy, the gold standard for diagnosing MAFLD, is invasive and resource-intensive; ultrasound and other imaging modalities show variable accuracy; and non-invasive serum biomarkers such as triglyceride–glucose index (TyG), triglyceride–glucose–body mass index (TyG-BMI), triglyceride–glucose–waist circumference (TyG-WC), and fatty liver index (FLI) remain understudied in many low- and middle-income settings [4]. In the Vietnamese cross-sectional study conducted at the Health Screening Department of University Medical Center Ho Chi Minh City between September 2024 and January 2025, 290 adults undergoing routine check-ups with abdominal ultrasonography were enrolled, of whom 32.76% were diagnosed with MAFLD using accepted criteria [4]. Patients with MAFLD were older, more frequently male, and had significantly higher BMI, waist circumference, blood pressure, rates of metabolic comorbidities, and abnormal biochemical parameters than non-MAFLD participants [4].

The diagnostic performance was highest for TyG-BMI and FLI, both achieving AUROC = 0.89, followed by TyG-WC (0.88) and TyG (0.82) [4]. In gender-stratified analysis, all indices performed better in females than in males: TyG-BMI achieved the highest AUROC of 0.91 in women (cutoff 205.71, sensitivity 80%, specificity 90%), comparable to FLI (0.90); in males, TyG-BMI and FLI each reached an AUROC of 0.85 [4]. The authors identify TyG-BMI and TyG-WC as reliable alternatives to FLI for MAFLD screening and prediction, particularly suited for primary-care and community-based settings. They emphasize that this study fills a specific geographic evidence gap, given the limited prior application of serum biomarkers in Vietnam [4].

### 5.2. Homocysteine as a Pediatric Biomarker

Pediatric MASLD represents an increasingly recognized clinical challenge, addressed by Mosca and colleagues through the lens of homocysteine, a sulfur-containing amino acid whose elevated levels have long been associated with endothelial dysfunction, oxidative stress, and systemic inflammation [3]. In a retrospective observational study of 182 children with MASLD (of whom 89 underwent liver biopsy), Hcy proved to be independently associated with homeostatic model assessment for insulin resistance (HOMA-IR) (β = 0.55; *p* = 0.049), TG/HDL (β = 3.23; *p* = 0.002), and liver fibrosis (β = 2.59; *p* = 0.04), with a predictive accuracy of 81% for fibrosis [3]. A direct relationship was identified between Hcy and the histological grade of lobular inflammation and fibrosis (grade > 1), supporting a mechanistic link between Hcy and liver injury progression [3].

A threshold of >8 µmol/L enabled the identification of children with fibrosis while maintaining moderate sensitivity, and Hcy alone outperformed combinations with APRI, FIB-4, and TG/HDL (AUC 0.62, 0.63 and 0.69 respectively), while APRI achieved only 0.50 (uninformative) and FIB-4 0.67 (not statistically significant) [3]. These findings are consistent with prior cohort data showing that elevated Hcy is associated with an increased risk of MASLD and elevated liver enzyme levels, but that genetic liability to NAFLD does not influence Hcy levels—suggesting that the Hcy–MASLD relationship reflects systemic inflammation and oxidative stress rather than shared genetic etiology [3]. The TG/HDL ratio—an inexpensive and widely available marker of insulin resistance and atherogenic dyslipidemia—is highlighted as deserving further investigation in pediatric MASLD populations [3]. The authors propose that future interventions targeting hyperhomocysteinemia (e.g., vitamin supplementation) could modify the natural history of pediatric MASLD; however, prospective trials are required to validate this hypothesis [3].

### 5.3. Sociodemographic and Lifestyle Determinants at Population Scale

A complementary epidemiological contribution comes from López-González and colleagues, who assessed the relationship between sociodemographic variables (age, sex, socioeconomic status), healthy habits (smoking, Mediterranean diet adherence, and physical activity), and stress—measured by Cohen’s Perceived Stress Scale—using values of three MAFLD risk scales in a sample of 16,708 Spanish workers [8]. All analyzed variables were associated with scores on the three risk scales, with the strongest associations (as represented by odds ratios) observed for age and physical activity [8].

The profile of individuals at highest risk of elevated MAFLD risk-scale values was characterized by male sex, age of 50 or older, lower socioeconomic status (manual laborers—“blue collar”), smoking, sedentary behavior, low adherence to the Mediterranean diet, and high stress-scale scores [8]. The magnitudes were substantial: among men aged 50–69, the prevalence of high FLI, high Hepatic Steatosis Index, and high LAP was 31.3%, 55.8%, and 48.9%, respectively; among blue-collar workers, the corresponding figures were 21.5%, 48.5%, and 38.3%, respectively, each significantly higher than those among white-collar workers [8]. Values increased with age, were higher in those with lower adherence to the Mediterranean diet and higher stress levels, and were uniformly lower in women—all differences were highly statistically significant (*p* < 0.001) [8]. The study provides strong population-level arguments for targeted lifestyle interventions against sedentary behavior and for the promotion of Mediterranean diet adherence, particularly in older male manual workers; this is a high-yield preventive strategy that operates precisely on the modifiable determinants identified by the Special Issue’s editorial frame.

## 6. The Pediatric and Adolescent Axis—Sarcopenia and NAFLD Severity

The diagnostic and clinical challenges of pediatric MASLD extend beyond biomarker discovery to include body-composition phenotypes. Kwon and colleagues retrospectively studied 122 patients aged 13–18 years diagnosed with both NAFLD and sarcopenia, using laboratory tests, abdominal ultrasonography, and multifrequency bioelectrical impedance analysis [6]. The authors stratified sarcopenia into tertiles based on the skeletal muscle-to-fat ratio, assessed NAFLD severity by ultrasonography, and measured cardiometabolic risk using the TyG index and the atherogenic index of plasma [6].

Patients in the lower MFR tertiles—those with more severe sarcopenia—exhibited greater NAFLD severity on ultrasound (*p* < 0.001) and significantly higher TyG and atherogenic index of plasma (AIP) values [6]. MFR showed an independent, negative correlation with NAFLD severity (*p* < 0.001) [6]. MFR also negatively correlated with TyG (r = −0.232, *p* = 0.011) and AIP (r = −0.222, *p* = 0.016), and the inverse relationship persisted even after accounting for BMI, underscoring the independent contribution of sarcopenia to NAFLD severity [6]. These findings extend prior work by demonstrating that individuals with sarcopenia are more likely to have severe NAFLD and that lower skeletal muscle indices are linked to advanced fibrosis [6]. Sarcopenia, once considered a disease of older adults, is now observed even in relatively young individuals—especially as obesity rates increase—and the coexistence of increased fat mass and reduced muscle mass (sarcopenic obesity) merits particular clinical attention in adolescents [6]. The practical implication is that resistance training aimed at increasing muscle mass should be considered a therapeutic component for NAFLD and cardiometabolic disease in adolescents with sarcopenia—a recommendation that the authors pair with calls for strengthening physical activity education in this age group [6].

## 7. The Genetic and Immunological Axis—Polymorphisms in MASLD

At the molecular level, Coste and colleagues addressed a different facet of the Special Issue’s call for pathomechanism data. Their exploratory prospective study evaluated a potential association between IL17A/F gene polymorphisms and MASLD in a Romanian patient population, recognizing that IL17 plays a critical pathogenetic role connecting systemic inflammation with liver fibrosis—as previously demonstrated in the link between psoriasis and MASLD [2]. A total of 43 patients with MASLD (classified as MASH or MAFL) and 38 healthy controls from Cluj-Napoca, Romania, were genotyped by PCR-RFLP for four polymorphisms—IL17F-A7488G, IL17A-G197A, TLR4-Asp299Gly, and TLR4-Thr399Ile. Serum levels of IL17A, IL17F, and TLR4 were measured by ELISA [2].

Patients with MASLD exhibited significantly higher serum IL17A (median 108.9 vs. 76.2; *p* = 0.007), IL17F (median 15.2 vs. 7.3; *p* = 0.008), and TLR4 than controls [2]. Patients carrying variant genotypes had significantly increased odds of MASH—specifically (A/G + G/G) of IL17-A7448G (OR = 5.25), (G/A + A/A) of IL17-G197A (OR = 10.57), (Asp/Gly + Gly/Gly) of TLR4-Asp299Gly (OR = 3.52), and (Thr/Ile + Ile/Ile) of TLR4-Thr399Ile (OR = 9.87) [8]. Haplotype analysis indicated that the A/A haplotype of IL17 (A7448G, G197A) is a risk factor for MASLD, while no TLR4 haplotypes emerged as risk factors [2]. The study is among the first to evaluate IL17A/F polymorphisms specifically in a Romanian MASLD population (prevalence approximately 20% in Romania, 25–26% in Europe), and—although exploratory and limited by sample size and single-center design—it provides a basis for further association studies and potential application of inflammatory markers in MASLD diagnosis and risk stratification [2].

The mechanistic picture is best interpreted by integrating the Romanian genetics data with the Mexican Th2-RAS findings [7]. Both studies emphasize that the immune–metabolic interface is central to MASLD pathogenesis and its cardiovascular complications, but the immune phenotypes involved appear distinct (Th17/innate immunity is driven by TLR4 in one study; Th2 humoral immunity drives RAS modulation in the other). This duality suggests that MASLD may encompass distinct immunological endotypes with potentially different therapeutic targets—a hypothesis that should drive future single-cell and transcriptomic work in liver-tissue biopsy series [2,7].

## 8. The Therapeutic Axis—From Pharmacology to Natural Compounds

### 8.1. Pharmacological Convergence Between MASLD and AF

The Morawska review synthesizes a substantial body of evidence for pharmacological interventions that act on both the liver and the heart [1]. GLP-1 RAs—specifically dulaglutide, exenatide, liraglutide, and semaglutide—improve hepatic steatosis and reduce liver enzyme levels and have shown promise in reducing AF burden [1]. SGLT-2 inhibitors, statins, and thiazolidinediones have similarly demonstrated synergistic effects [1]. Statin use has also been linked to a reduced risk of MASLD progression to high-risk fibrosis (HR 0.60–0.61 in retrospective cohorts) [1]. The approval of resmetirom (the first FDA-approved drug for MASH and associated fibrosis) marks a clinical milestone but remains insufficient on its own, as patients with MASH—particularly those with advanced fibrosis—face an elevated long-term risk of progression to cirrhosis and liver-related events [1]. The therapeutic landscape therefore combines disease-specific antifibrotic agents (resmetirom), cardiometabolic agents with hepatic benefits (GLP-1 RAs, SGLT-2 inhibitors), and broadly acting cardiovascular protectants (statins)—each offering partial coverage of the MASLD–CVD axis [5].

### 8.2. White Tea in a Preclinical Obesity Model

Complementing the pharmacological perspective, Yılmaz and colleagues explore the therapeutic potential of natural antioxidant compounds in obesity, an upstream driver of MASLD and the related cardiometabolic spectrum [9]. Obesity—affecting over 600 million people globally and declared an epidemic by the World Health Organization—is responsible for numerous severe health conditions, particularly T2DM and metabolic syndrome [9]. The study used 72 male Sprague-Dawley rats randomized into nine groups of eight, with case groups receiving white tea alongside a high-fat diet, and a positive control group receiving orlistat with the HFD by oral gavage [9]. Serum leptin, asprosin, and insulin levels were measured by ELISA.

White tea administration led to a significant decrease in body weight, serum leptin and asprosin levels, glucose, total cholesterol, low-density lipoprotein cholesterol (LDL-C), and TBARS levels, as well as in HOMA-IR, and importantly, white tea also increased total testosterone levels in rats fed a high-fat diet (*p* < 0.05) [9]. Histological observations in adipose tissue—including the number and size of adipocytes—were also improved [9]. Mechanistically, white tea possesses higher total polyphenol and catechin concentrations than other teas (notably EGCG—epigallocatechin-3-gallate), conferring exceptional antioxidant potential; prior in vivo work has shown that EGCG decreases food consumption and blood leptin resistance while promoting energy expenditure and fat oxidation [9]. The implication is that natural compounds with strong antioxidant properties and minimal side effects, such as white tea, can mitigate the detrimental consequences of obesity—including improvements in insulin resistance and metabolic parameters—opening an adjunctive therapeutic avenue relevant to MASLD prevention in the general population.

## 9. Cross-Cutting Themes—A Synthesis

### 9.1. MASLD as a Systemic Disease Requiring Multidisciplinary Care

Every contribution in this Special Issue reinforces its central editorial thesis that MASLD is a systemic disease requiring multidisciplinary attention. From the hepatology–cardiology interface (Morawska et al. [1]; Różycka-Kosmalska et al. [5]) and the hepatology–nephrology interface (the PermAF cardio-renal phenotype described by Różycka-Kosmalska et al. [5]) to the hepatology–immunology interface (Coste et al. [2]; Méndez-García et al. [7]), pediatric hepatology (Mosca et al. [3]; Kwon et al. [6]), and lifestyle medicine (López-González et al. [8]; Yılmaz et al. [9]), the boundaries of traditional hepatology are repeatedly exceeded. The practical consequence is that MASLD patients must be assessed not only for liver-related complications but also for cardiovascular risk (including AF and HA), renal function, sarcopenia, metabolic inflammation, and modifiable lifestyle factors. Conversely, patients with AF, HA, or sarcopenic obesity should be screened for MASLD.

### 9.2. Tools That Work Differently in Different Populations

A subtle but consistent pattern emerges from the diagnostic studies: non-invasive tools perform best when calibrated to the population in which they will be deployed. The Vietnamese study shows that TyG-BMI and FLI achieve AUROC 0.89 in a Southeast Asian cohort, with all indices performing better in females than in males [4]. The Spanish study confirms that risk-scale values are uniformly lower in women than in men, and rise steeply with age, lower Mediterranean diet adherence, and higher stress levels [8]. The Romanian genetics study illustrates that the OR values associated with IL17 and TLR4 polymorphisms may be population-specific, and the pediatric biomarker study shows that even well-established indices like APRI and FIB-4 may lack predictive power in younger populations [2,3]. The clinical takeaway is that MAFLD/MASLD risk stratification is intrinsically demographic-dependent, and the field must move toward locally validated, population-specific screening algorithms rather than universal cutoffs.

### 9.3. The Role of Age as a Confounder

Age emerges as a unifying confounder across multiple analyses. The PermAF subgroup is dominated by a cardio-renal phenotype in which FIB-4 is elevated, but this likely reflects age and comorbidity rather than hepatic fibrosis per se [5]. The Spanish workers’ study demonstrates that risk-scale prevalence rises steeply between ages 50–69 compared to younger groups [8]. The Vietnamese study demonstrates that older age is associated with higher MAFLD prevalence [4]. In pediatric cohorts, age-specific reference ranges and developmental physiology must be considered when interpreting biomarkers such as Hcy and TyG [3,6]. Across all studies, the consistent methodological lesson is that MASLD biomarkers—whether serological, anthropometric, or imaging-based—must be interpreted in their age and comorbidity context.

### 9.4. Modifiable Determinants Versus Fixed Risk Factors

A practical contrast emerges between fixed and modifiable determinants of MASLD/CVD risk. Genetic polymorphisms (IL17, TLR4) [2], male sex [8], older age [4,5,8], and underlying cardio-renal comorbidity [5] represent fixed or difficult-to-modify factors. By contrast, Mediterranean diet adherence, physical activity, smoking, stress management, body composition (including sarcopenia), and metabolic control through pharmacological or natural agents (white tea, GLP-1 RAs, statins) all represent modifiable targets [1,6,8,9]. The López-González study quantifies the magnitude of the lifestyle contribution at the population level [8], and the Morawska review quantifies the magnitude of the pharmacological contribution (semaglutide reducing AF by 42% across 12,651 patients) [1]. The combined evidence argues that the greatest reduction in MASLD-associated cardiovascular morbidity is likely to come from combined interventions on multiple modifiable fronts, supported by targeted pharmacology in high-risk subgroups.

### 9.5. The Quantitative Anchoring of the Special Issue

Finally, the Special Issue is held together by a small number of quantitative anchors that recur across contributions:

- MASLD prevalence ~30% of adults globally; ~75% among those with overweight [1].
- NAFLD prevalence 42.2% in non-valvular AF cohorts [1].
- NAFLD OR for prevalent AF ~2.07 (nine studies, >360,000 subjects) [1].
- MASLD HR for incident AF 1.20 (16 studies, ~19.5 million subjects, 7.2-year follow-up) [1].
- Semaglutide reduces AF incidence by 42% (10 RCTs, 12,651 participants, ~68-month follow-up) [1].
- Resmetirom achieves 25.9–29.9% MASH resolution vs. 9.7% placebo (phase 3, n = 966) [1].
- Statin HR for MASLD fibrosis progression 0.60–0.61 (n = 1238) [1].
- MASLD prevalence in AF subtypes: 47%/51%/27% (PAF/PeAF/PermAF) [5].
- TG/HDL inversely correlated with AF progression (3.32 vs. 1.93) [5].
- TyG-BMI AUROC 0.91 in Vietnamese women (cutoff 205.71) [4].
- Hcy predictive accuracy 81% for pediatric fibrosis (n = 182) [3].
- MFR tertiles independently predict adolescent NAFLD severity (n = 122) [6].
- IL17-G197A G/A + A/A OR 10.57 for MASH (n = 43 Romanian MASLD) [2].
- Strongest lifestyle risk factors: age and physical activity (n = 16,708 Spanish workers) [8].
- White tea reduces HOMA-IR and increases testosterone in HFD rats (n = 72) [9].
- Th2 cytokines (IL-4, IL-13) are best ACE predictors in Mexican MASLD liver biopsies [7].

These anchors can be reused in the design of future clinical trials, validation studies, and meta-analyses as quantitative benchmarks for power calculation and effect-size estimation.

## 10. Open Questions for Future Research

- Causal versus associative relationships. The Morawska review surfaces a persistent debate about whether MASLD is an independent causal risk factor for AF or a marker of shared cardiometabolic risk [1]. Future Mendelian randomization studies with instrumental variables capturing hepatic versus metabolic pathways could disentangle these possibilities.
- Optimal treatment sequencing. The expanding therapeutic armamentarium (statins, GLP-1 RAs, SGLT-2 inhibitors, resmetirom) raises the practical question of which agent to initiate, in which order, and in which combination—a question unanswered by current evidence [1]. Pragmatic head-to-head trials and adaptive-design comparison studies represent the most efficient route to a definitive answer.
- Biomarker validation in elderly populations. The predictive value of FIB-4 and similar indices may degrade in elderly populations with cardiorenal comorbidity, requiring recalibration or alternative biomarkers [5]. Specific cutoffs for octogenarians and nonagenarians should be derived from prospective cohorts.
- Long-term cardiovascular outcomes of MASLD-specific therapies. While resmetirom has demonstrated MASH-resolution efficacy, its long-term effect on cardiovascular events is unknown; the same gap applies to the next generation of MASH drugs under development [1]. Extension trials and cardiovascular endpoint studies with at least five-year follow-up are essential.

Hcy as a therapeutic target in pediatric MASLD. The Mosca et al. findings highlight the association of Hyc with cardiometabolic risk in pediatric MASLD, warranting future perspective studies to establish its clinical utility [3]. Randomized vitamin-supplementation trials with paired liver-biopsy endpoints in children with hyperhomocysteinemia should be prioritized.

Mechanistic dissection of the IL17 axis. The Coste et al. data identify IL17 and TLR4 polymorphisms as MASLD risk modifiers, but the cellular and molecular pathways (e.g., neutrophil extracellular trap formation, Th17 polarization, and gut–liver axis perturbations) remain to be systematically traced in this patient population [2]. Single-cell sequencing of liver biopsies stratified by genotype is a concrete next step.

Integration of the Th2-RAS axis into HA management. The Méndez-García findings identify IL-13 as differentially expressed in MASLD patients with SAH and correlated with hepatic ACE, opening the question of whether Th2 modulation could alter blood pressure control in this group [7]. Pharmacological modulators of IL-13/STAT6 should be evaluated in proof-of-concept trials.

Translating sarcopenia interventions to adolescent NAFLD outcomes. The Kwon et al. data establish a relationship between MFR and NAFLD severity in adolescents—but whether resistance-training interventions can reverse this relationship remains untested [6]. School-based or clinic-based resistance-training RCTs in adolescents are feasible and underpowered evidence already exists to justify them.

Optimal Mediterranean diet and physical activity protocols for MAFLD prevention. The López-González data establish risk factor magnitudes but do not specify optimal intervention protocols, durations, or delivery modes [8]. Comparative effectiveness studies of different Mediterranean diet prescriptions and physical activity regimens (including dose, modality, and intensity) are needed.

Clinical translation of white tea findings. The Yılmaz et al. data must be followed by clinical trials in humans, with attention to dosing, formulation, and long-term safety [9]. Phase 1/2 trials with metabolic and cardiovascular biomarkers represent the next translational milestone.

## 11. Extended Synthesis—MASLD and Cardiovascular Disease in the 2025–2026 Evidence Base

### 11.1. The Epidemiological Anchor

The editorial presented above anchored its quantitative estimates on the classical “approximately 30% of the global adult population” figure [1]. Within the past 24 months, several authoritative updates have converged on a higher and more granular picture. Younossi, Kalligeros, and Henry establish that 38% of all adults and 7–14% of children and adolescents currently meet MASLD criteria, with a projected adult prevalence of more than 55% by 2040—making MASLD a disease affecting more than half of the global adult population within one generation [10]. Le and colleagues’ hierarchical Bayesian forecast arrives at a strikingly similar 2040 projection of 55.2% global NAFLD prevalence, with the highest estimates (above 60%) clustered in Asia and Europe [11]. Teng and colleagues’ systematic review documents the trajectory: NAFLD global prevalence 32% (40% in males, 26% in females) in pooled analysis, having risen from 26% in studies from 2005 or earlier to 38% in studies from 2016 or beyond, with prevalence exceeding 40% in the Americas and South-East Asia [12]. A 2024 analysis by Torre and colleagues reports pooled MASLD prevalence of 38.77%, with the highest in Europe (55.33%), followed by Asia (36.31%) and North America (35.99%) [13]. MASLD has now become the leading indication for liver transplantation in the United States among women and among patients with hepatocellular carcinoma, but—as Younossi emphasizes—the most common cause of mortality in MASLD patients remains cardiovascular disease, additionally associated with de novo T2DM, CKD, sarcopenia, and extrahepatic cancers [10].

### 11.2. Meta-Analyses and the Quality of Evidence

Within the editorial’s framework, the question of whether MASLD acts as an independent cardiovascular risk factor or as a marker of underlying cardiometabolic dysfunction remains unresolved [1]. Recent meta-analyses sharpen this debate. A 2025 systematic review with meta-analysis by Hung and colleagues pooled 19 longitudinal cohort studies encompassing approximately 1.25 million participants followed for a median of 8.4 years, demonstrating that MASLD is associated with elevated risk of incident cardiovascular events; after adjustment for age, sex, and cardiometabolic factors, the association remained significant but was substantially attenuated (OR 1.62; 95% CI 0.06–3.18; *p* = 0.043), supporting the conclusion that a substantial proportion of the apparent MASLD–CVD association is mediated by shared cardiometabolic risk factors [14]. The earlier Mantovani meta-analysis had already documented a graded relationship between disease severity and cardiovascular risk, with NAFLD increasing the risk of both fatal and non-fatal CVD events and the magnitude of risk rising with fibrosis stage [15]. The Bhatt systematic review of NAFLD/MASLD as a cardiometabolic fuel consistently supports this picture [16]. The synthesis emerging across 2025 reviews is therefore moving away from the framing “MASLD is an independent CV risk factor” toward the more nuanced interpretation “MASLD identifies a high-risk cardiometabolic phenotype”—a model that aligns with Mendelian randomization evidence and with the phenotype-stratified data presented in the editorial.

The emerging entity MASLD + moderate alcohol intake (MetALD) requires separate consideration. In a 2024 systematic review by Ciardullo and colleagues encompassing five studies and 9,824,047 participants, MetALD was associated with elevated cancer-related mortality (HR 2.10; 95% CI 1.35–3.28) and cardiovascular mortality (HR 1.17; 95% CI 1.12–1.22) above the levels seen in MASLD alone [17]. Kim and colleagues’ Korean nationwide data corroborate this hierarchy, reporting CVD hazard ratios of 1.43 for MAFLD versus 1.09 for NAFLD, with fatty liver disease overall associated with HR 1.56 [18]—a finding interpreted by the authors as supporting the prognostic value of metabolically anchored definitions, although the metabolically dysregulated features themselves likely contribute to the higher mortality.

### 11.3. The Liver–Heart Axis and Its Embedding in the 2026 CKM Framework

The 2026 AHA/ACC/ADA/ASN Guideline for the Prevention, Detection, Evaluation, and Management of cardiovascular–kidney–metabolic syndrome formalizes a five-stage (Stages 0–4) CKM construct that integrates hepatic parameters into the cardiovascular risk algorithm, addressing the persistent gap noted in the editorial’s Section 1.2 on the shortage of data linking MASLD to cardiovascular events through shared risk pathways [19]. Theodorakis and Nikolaou argue that this framework should be further extended to a cardiovascular–renal–hepatic–metabolic syndrome, recognizing inflammation, oxidative stress, endothelial dysfunction, and renin–angiotensin–aldosterone system activation as common pathophysiological drivers [20]. Lee, in a 2026 commentary in *Journal of Diabetes Investigation*, notes that hepatic fat accumulation precedes the development of insulin resistance by 5–7 years and that the liver–heart axis, originally constructed around hepatic biomarkers, must now be embedded into the broader CKM risk-assessment infrastructure [21]. Khan and colleagues’ 2025 *JACC* primer for cardiologists emphasizes that the 2022 AHA Scientific Statement already formally recognized NAFLD as an independent ASCVD risk factor and that absolute LDL-C reductions correlate with lower major CV event rates, particularly in patients with elevated aminotransferases [22].

### 11.4. Mechanistic Pathways Linking MASLD to CVD

#### 11.4.1. Genetic and Mendelian Randomization Evidence

The editorial’s Section 4.1 noted that the genetic architectures of AF (PITX2, KCNN3, TTN, MYH6) and of hepatic steatosis (PNPLA3, TM6SF2) are largely non-overlapping, supporting a primarily systemic–metabolic rather than heritable shared pathway [1]. Updated 2024–2025 Mendelian randomization studies consolidate this picture. Wang and colleagues, in a two-sample Mendelian randomization analysis using the GK–GKRP axis as an instrumental variable, demonstrate that genetically proxied impaired GK–GKRP interaction causally increases plasma triglycerides, LDL-C, and ApoB, decreases HDL-C, and elevates the risks of both MASLD and coronary artery disease, validating a lipid-mediated causal pathway [23]. Zhou and colleagues further refine the genetic picture by documenting that the protective effect on coronary artery disease (CAD) seen with PNPLA3/TM6SF2 variants may be mediated through differential effects on lipid metabolism [24]. These findings align with the role of PNPLA3/TM6SF2 in hepatic lipid handling and reinforce the argument—emphasized in the editorial and re-stated by Driessen and colleagues [25]—that MASLD most likely contributes to atherosclerosis via mixed hyperlipidemia rather than via liver-derived procoagulant mediators, although the latter hypothesis remains insufficiently supported by current genetic evidence.

#### 11.4.2. Inflammation, Hepatokines, and HFpEF Phenotypes

Mustafa and colleagues [26] and Suresh and colleagues [27] expand the mechanistic picture available to the editorial’s readers beyond the AF-anchored view of Morawska and colleagues [1]. They emphasize that MASLD interacts with the cardiovascular system through insulin resistance, chronic low-grade inflammation, endothelial dysfunction, atherogenic dyslipidemia, dysregulated hepatokines (e.g., fetuin-A), gut-derived metabolites, and genetic determinants, with risk rising in step with hepatic disease severity and fibrosis stage [24,26,27]. Ismail and colleagues conclude that MASLD seems to be an independent CV risk factor, but caution that further studies are needed to clarify the distinct contributions of MASLD stages and their interplay with the metabolic syndrome [28]. Basta and colleagues’ 2026 framework review integrates this picture with the therapeutic implications [29].

The editorial focused on atrial fibrillation as the principal cardiovascular endpoint of MASLD [1]. Newer 2025 evidence extends the cardiovascular phenotype to HF. Chang and colleagues demonstrate that MASLD—but not other SLD subtypes—is associated with elevated risk specifically of HFpEF [30]. Salah and colleagues previously proposed three NAFLD-related HFpEF phenotypes—obstructive HFpEF, metabolic HFpEF, and advanced liver fibrosis HFpEF—and recommended that NAFLD-related HFpEF be recognized as a distinct clinical entity [31]. Yang and colleagues describe the mechanism: in advanced NAFLD, spontaneous portosystemic and arteriovenous shunts form, producing a hyperdynamic circulation with elevated resting cardiac output but limited preload reserve, which manifests as HFpEF under stress [32]. Together, these findings substantially extend the cardiovascular phenotype sketched in the editorial beyond AF and ASCVD.

#### 11.4.3. Triglyceride–Glucose Indices as Integrated Risk Markers

The editorial’s Section 5 discussed TyG-related indices as non-invasive diagnostic tools [4]. New 2025 evidence documents their prognostic value in patients with established MASLD. Qiao and colleagues’ analysis in UK Biobank participants with MASLD demonstrates that TyG, TyG-BMI, TyG-WC, and TyG-WHtR are positively associated with the risk of incident overall CVD, CHD, all-cause mortality, and cardiovascular mortality, with hazard ratios of 1.19–1.39 across indices for overall CVD, and TyG-WHtR/TyG-WC showing the highest predictive performance [33]. These findings integrate MASLD and CVD risk in a single quantitative framework that is well suited to primary-care and population-scale screening.

### 11.5. Clinical Phenotypes Across the Cardiovascular Spectrum

The editorial’s contribution by Różycka-Kosmalska, Luzak, and Kosmalski documented a phenotypic gradient across paroxysmal, persistent, and permanent AF, with MASLD prevalence of 47.1%/51.4%/27.1%, respectively [5]. The 2025–2026 literature extends this view. Suresh and colleagues’ state-of-the-art review consolidates the contemporary evidence that MASLD patients present with elevated risk of arrhythmia (including AF), atherosclerotic heart disease, heart failure, and cardiovascular mortality [27]. Khan and colleagues’ 2025 primer for cardiologists notes that the three principal clinical pathways through which MASLD enters the cardiovascular domain are ischemic heart disease, arrhythmias (especially AF), and heart failure [22]. Driessen and colleagues, in their comprehensive *Hepatology* review, note that despite increased atherosclerosis in MASLD patients no consistent increase in ASCVD mortality has been demonstrated—adding nuance to the editorial narrative [25]. Manoria and Manoria frame MASLD as a gateway for ASCVD requiring early intervention and multidisciplinary care, emphasizing that cardiovascular mortality already exceeds liver-related mortality in MASLD patients [34].

A particularly important contemporary insight is that atherogenic dyslipidemia is the dominant shared mechanistic substrate. Jamalinia and Lonardo note that MASLD was specifically associated with severe CAC > 300 (aOR 1.38) [35]. Khan and colleagues’ primer underscores that statin therapy is safe and recommended in MASLD patients, with absolute CV benefits greatest among those with elevated aminotransferases [22]—providing a quantitative foundation for the lipid-lowering approach already anchored in the editorial’s Quantitative Anchors Section. The intersection of MASLD with the broader cardiovascular disease spectrum has been quantified in large cohort studies: in a Danish population, Kyhl and colleagues document a strong graded relationship between MASLD severity and cardiometabolic comorbidities [36], while Bhatt and colleagues in their systematic review demonstrate that NAFLD/MASLD fuels cardiovascular risk across multiple cohorts [16].

### 11.6. Special Populations—Lean MASLD and Geographic Variation

The editorial documented the strong male–female gradient in MASLD risk [8] and the higher prevalence among Vietnamese populations (32.76%) [4]. Two newly relevant dimensions emerge from the 2025–2026 literature: the lean MASLD phenotype and differential geographic progression.

Mapouka and colleagues’ 2026 systematic review and meta-analysis of 31 studies encompassing 10,735,550 participants provides the most rigorous quantitative picture: non-lean MASLD individuals have higher odds of liver fibrosis (pOR 2.0; 95% CI 1.0–3.9; *p* = 0.04) and hepatic steatosis (pOR 2.1; 95% CI 1.5–2.9; *p* < 0.001), whereas lean MASLD individuals have lower odds of cirrhosis (pOR 0.7; 95% CI 0.5–0.9; *p* = 0.007) and HA (pOR 0.7; 95% CI 0.6–0.9; *p* = 0.002). Critically, no statistically significant difference between lean and non-lean MASLD was observed for mortality (pOR 1.4; 95% CI 1.0–2.0; *p* = 0.06), MASH (pOR 1.2; 95% CI 0.7–2.0; *p* = 0.5), or cardiovascular disease (pOR 0.9; 95% CI 0.7–1.0; *p* = 0.1) [37]. Lebovics, Frishman and colleagues’ 2025 review highlights that lean MASLD patients exhibit distinctive metabolic, genetic, and microbiome profiles—visceral adiposity, sarcopenia, hepatic fibrosis, systemic inflammation, and endothelial dysfunction—converging toward elevated CV risk despite normal BMI [38]. Fouad and colleagues document similar patterns specifically for lean MASH, where the cardiometabolic burden is particularly striking [39]. These data require that screening for MASLD and associated CV risk be expanded beyond obesity-based metrics.

Geographically, the integration of MASLD with the CKM framework has been quantified in CKM-stage-stratified cohorts. Liang and colleagues’ 2025 analysis demonstrates a strong graded association: relative to CKM Stage 0, MASLD prevalence rates ascend progressively across Stages 1–4, with adjusted odds ratios reaching 8.34/7.38 for CKM Stage 4 versus Stage 0 [40]. Zheng and colleagues’ 2026 study confirms that CKM stage is significantly associated with MASLD prevalence, advanced liver fibrosis, and cirrhosis [41]. Wan and colleagues’ cross-national comparison demonstrates that MASLD is associated with a 1.708-fold increased risk of advanced CKM in the USA and a 2.061-fold increased risk in China [42].

### 11.7. Real-World Evidence, Public Health, and Future Research

Huang, Hewitt, Li, and Alazawi’s 2025 *Lancet Regional Health—Europe* review synthesizes the role of real-world evidence in MASLD research [43]. RWE complements RCTs by providing information from larger, more diverse populations, longer follow-up, and real-world drug utilization patterns. In MASLD, RWE reveals substantial regional epidemiologic variation and is used to refine patient stratification and clinical trial design. The authors point to artificial intelligence and multimodal data integration as the principal future direction—a frontier that the editorial’s Section 1.4 implicitly anticipates in its call for “Large, multicenter, prospective validation” [1].

Adinolfi, Marrone, Izzi, and Craxì’s 2026 review frames the contemporary therapeutic landscape, with particular emphasis on the decompensating role of MASLD when it coexists with T2DM or CKD, both of which dramatically worsen clinical outcomes and require multifaceted management strategies [44]. Tilg, Petta, Stefan, and Targher’s 2026 JAMA review provides a definitive summary of MASLD in adults, noting that approximately 99% of NAFLD patients meet MASLD criteria, and up to 70–90% of patients with obesity or T2DM have MASLD, with stage of liver fibrosis as the strongest disease-specific predictor of extrahepatic complications including CVD, T2DM, and CKD [45].

### 11.8. Therapeutic Innovations—Randomized Trial Evidence from 2024 to 2025

#### 11.8.1. Resmetirom—The First FDA-Approved MASH Drug

The editorial presented resmetirom as the first FDA-approved drug for MASH with associated liver fibrosis, with MAESTRO-NASH results showing 25.9–29.9% MASH resolution (vs. 9.7% placebo) and 24–26% fibrosis improvement (vs. 14% placebo) at 52 weeks [1]. The 2024 AASLD Practice Guidance update by Chen and colleagues formalizes clinical implementation: resmetirom received accelerated FDA approval in March 2024 for adults with MASH and F2–F3 fibrosis [46]. The AASLD writing group provides implementable guidance on patient selection (using non-invasive assessments), concomitant therapy (maximum rosuvastatin/simvastatin 20 mg/d, atorvastatin/pravastatin 40 mg/d when used with resmetirom), safety monitoring (withdrawal thresholds defined), and assessment of efficacy and futility at 12 months [46]. Importantly, Harrison and colleagues’ MAESTRO-NASH data confirmed that resmetirom produces reductions in atherogenic lipids and lipoproteins—including LDL-C, apoB, triglycerides, apoCIII, lipoprotein(a), and VLDL cholesterol—without affecting THR-α activity in the heart and bone [47]. These lipid effects position resmetirom as a uniquely dual-acting agent, simultaneously addressing hepatic fibrosis and a component of the cardiometabolic risk profile.

#### 11.8.2. Semaglutide—The ESSENCE Phase 3 Trial

The ESSENCE trial—the first completed phase 3 trial of semaglutide in MASH—was reported in 2025 publications. Newsome, Sanyal, Kliers, and colleagues provided the Part 1 (week 72) analysis of the first 800 participants with biopsy-defined F2/F3 MASH, randomized 2:1 to once-weekly subcutaneous semaglutide 2.4 mg or placebo [48]. The primary results demonstrated that resolution of steatohepatitis with no worsening of fibrosis was achieved by 62.9% of semaglutide recipients vs. 34.3% on placebo (estimated difference in responder proportions of 28.7%; 95% CI 21.1–36.2; *p* < 0.001), and improvement in fibrosis with no worsening of steatohepatitis was achieved by 36.8% vs. 22.4% (EDP 14.4%; 95% CI 7.5–21.3; *p* < 0.001) [48]. In the secondary analysis combining histology with non-invasive tests, 45.7% of semaglutide recipients met all disease-activity criteria vs. 10.4% on placebo, and 84.3% vs. 54.9% met at least one fibrosis-related criterion [48]. Sanyal, Newsome, Kliers, and colleagues’ definitive phase 3 publication concludes that once-weekly semaglutide at a dose of 2.4 mg improved liver histologic results in patients with MASH and moderate or advanced liver fibrosis [49]. Mademlis and colleagues’ editorial commentary positions the ESSENCE results as simultaneously addressing “the ESSENCE of cardio-metabolic health” [50].

#### 11.8.3. Combined and Sequential Pharmacological Strategies

Two years of post-marketing data and additional trial analyses provide partial clarification of which agent to initiate, in which order, and in which combination. Khan and colleagues’ 2025 primer for cardiologists emphasizes that statins are safe and recommended in MASLD, that GLP-1 RAs and SGLT-2 inhibitors should be initiated in patients with T2DM and/or HF/CKD regardless of MASH activity, and that combined interventions should explicitly target Mediterranean diet, weight reduction of 7–10% for MASH/fibrosis regression, and 150–200 min per week of moderate aerobic activity with avoidance of alcohol [22]. Chang and Lee’s post hoc analysis demonstrates that the efficacy of resmetirom for MASH endpoints was not impaired by stable concomitant use of GLP-1 RA or SGLT2i. This is clinically important given that approximately 40% of T2DM participants in MAESTRO-NASH still had MASH despite prior GLP-1 RAs or SGLT2i use, suggesting that drug combinations may be necessary for full MASH control [51].

### 11.9. Updated Quantitative Anchors

Extending the editorial’s quantitative anchoring framework (Section 9.5), the present synthesis adds the following 2025–2026 numerical anchors:

▪ MASLD global prevalence: 38% of adults, 7–14% of children/adolescents; projected >55% in 2040 [10,12,13].
▪ MASLD prevalence by region: Europe 55.33%, Asia 36.31%, and North America 35.99% [13].
▪ MAFLD vs. NAFLD CVD HR: 1.43 vs. 1.09 [18].
▪ MetALD CV mortality: HR 1.17; 95% CI 1.12–1.22; cancer mortality HR 2.10; and 95% CI 1.35–3.28 [17].
▪ Semaglutide ESSENCE week 72 results (F2/F3 MASH):
  ▪ MASH resolution: 62.9% vs. 34.3%; EDP 28.7% [48,49].
  ▪ Fibrosis improvement: 36.8% vs. 22.4%; EDP 14.4% [48,49] All-criteria response (ALT + FAST + histology): 45.7% vs. 10.4% [48].
▪ Resmetirom MAESTRO-NASH week 52 results (MASH + F1b–F3): 26–30% MASH resolution vs. 10% placebo; 24–26% fibrosis improvement vs. 14% placebo; and reduces LDL-C, apoB, TG, apoCIII, lipoprotein(a) and very low-density lipoprotein cholesterol [47].
▪ CKM stage-stratified MASLD OR (Stage 4 vs. Stage 0): 8.34/7.38 [40].
▪ Severe liver disease OR across CKM stages: rising to OR 3.79 [40].
▪ TyG-WHtR/TyG-WC hazard ratios for incident CVD in MASLD: 1.19–1.39 [33].
▪ Lean vs. non-lean MASLD: pooled OR for CVD 0.9; 95% CI 0.7–1.0; and fibrosis 2.0 (non-lean higher) [37].
▪ MASLD vs. other SLD: HFpEF risk is specifically elevated in MASLD [30].
▪ MASLD → CAD causal pathway via GK-GKRP disruption: TG, LDL-C, and ApoB are all causally elevated; HDL-C is decreased [23].
▪ Severe CAC (>300) association with MASLD: aOR 1.38 [35].
▪ MASLD is associated with incident T2DM, CKD, sarcopenia, and extrahepatic cancers [10].

### 11.10. Clinical Implications for Cardiologists Updated to 2026

Integrating the 2025–2026 evidence base, the following updated clinical conclusions complement the editorial’s synthesis:

1. MASLD has reached “epidemiological maturity”: 38% of adults currently, projected >55% by 2040 [10,12,13]—making MASLD a routine component of cardiovascular risk assessment rather than a specialist referral diagnosis.
2. MASLD identifies a high-risk cardiometabolic group: The 2025 meta-analytic evidence indicates that a substantial proportion of the apparent MASLD–CVD association is mediated by shared cardiometabolic risk factors [14,15]. The consensus is moving toward treating MASLD as a high-risk stratification marker.
3. Fibrosis is the principal amplifier: F2–F3 fibrosis stages are associated with proportionally higher CVD risk, supporting the integration of non-invasive fibrosis assessment into CKM risk algorithms [19,44,45].
4. HFpEF is a distinct MASLD phenotype: Recent evidence identifies MASLD-specific HFpEF, with three proposed phenotypes (obstructive, metabolic, advanced liver fibrosis) [30,31,32]—providing the mechanistic basis for the editorial’s Section 4.2 observation of 48.6% HFpEF prevalence in PermAF patients [5].
5. Drug-specific therapeutic innovations:
  - Resmetirom: First FDA-approved MASH drug; reduces atherogenic lipids beyond hepatic fibrosis [46,47].
  - Semaglutide 2.4 mg: MASH resolution of 62.9% vs. 34.3%, fibrosis improvement of 36.8% vs. 22.4% [48,49].
  - Combined resmetirom + GLP-1 RA: Efficacy preserved in post hoc analysis [51].
  - GLP-1/GIP co-agonists: tirzepatide phase 3 data show approximately 20% body weight loss (SURPASS and SURMOUNT-1 trials), positioning these agents as promising MASLD therapeutics [52].
6. Lean MASLD cannot be excluded by BMI alone: Pooled evidence shows similar CVD risk in lean and non-lean MASLD (pOR 0.9; not significant) [37,38].
7. CKM staging is the operational framework: Strong graded association between CKM stage and MASLD prevalence (OR ~7–8 for CKM Stage 4), supporting the unified CKM construct [19,40,41].
8. Lifestyle remains cornerstone, but trials now support drug combinations: Mediterranean diet, 7–10% weight loss, and 150–200 min/week of aerobic activity remain first-line, with resmetirom + GLP-1 RA combinations supported by AASLD 2024 guidance [22,46].
9. Geographic variation deserves locally calibrated screening algorithms. Prevalence differences in Europe (55.33%), Asia (36.31%), and North America (35.99%) require culturally tailored screening protocols [4,13].
10. Inflammation, oxidative stress, and lipid metabolism are shared mechanisms: Theodorakis and Nikolaou’s CRHM framework identifies these as common drivers, providing a pathophysiological basis for unified MASLD–CVD management [20].

By integrating these 2025–2026 quantitative anchors and the updated pathophysiological and therapeutic framework, the editorial’s narrative expands into a multisystem, fibrosis-staged, and therapeutically actionable view of MASLD as a central node in cardiometabolic medicine. The findings consolidate without contradicting the Special Issue’s original editorial thesis that MASLD management cannot be confined to hepatology and that a multidisciplinary framework integrating hepatology, cardiology, endocrinology, nephrology, immunology, lifestyle medicine, and primary care is required.

## 12. Conclusions

This Special Issue of *Life* advances a coherent narrative: MASLD is a global, systemic, and progressive disease whose principal health burden lies outside the liver—in the heart, the kidneys, the vasculature, the immune system, and the metabolic determinants of body composition. The contributions collected here move beyond the assertion of association to specify mechanisms (oxidative stress, profibrotic mediators, IL-17-driven inflammation, Th2–RAS crosstalk), quantify risk (MASLD prevalence of approximately 30% globally, rising to 38–55% in current and projected adult populations; AF HR 1.20 even after full adjustment; OR ~2.07 for prevalent AF; coronary artery disease, HA, and structural heart disease co-occurring at rates exceeding 42%), and propose intervention points (weight loss, Mediterranean diet, physical activity, sarcopenia screening and resistance training, GLP-1 RAs, SGLT-2 inhibitors, statins, resmetirom, semaglutide, and antioxidant-rich natural products).

By consolidating quantitative anchors from complementary perspectives—mechanistic, clinical–phenotypic, diagnostic, epidemiological, and therapeutic–pharmacological and nutritional—this Special Issue addresses seven distinct knowledge gaps identified in Section 1.2 and lays out a coordinated agenda of five thematic priority research directions for the field. The overarching clinical implication is that MASLD cannot be managed in isolation within hepatology. Treating the disease effectively requires a multidisciplinary framework integrating hepatology, cardiology, endocrinology, nephrology, immunology, lifestyle medicine, and primary care—a framework this Special Issue both exemplifies and advocates for.

The Special Issue is structured such that this editorial synthesis is the final publication in the collection, scheduled for publication only after each of the nine original submissions has completed the peer-review process. Accordingly, no further submissions will be accepted after this editorial is published, and the contributions summarized above constitute the definitive content of the *Life* Special Issue “Metabolic Health: The Interplay Between NAFLD, MAFLD, MASLD and Cardiovascular Disease.” Researchers interested in extending this agenda are encouraged to build on the gaps and priorities explicitly enumerated in Section 1.2, Section 1.4 and Section 11.2, thereby translating the synthesis presented here into the next generation of multicenter, population-validated, mechanistic, and interventional studies in MASLD and cardiovascular disease.

## Figures and Tables

**Table 1:** Alignment of the nine manuscripts contributed to this Special Issue with the seven knowledge gaps identified in Section 1.2, specifying for each submission the knowledge gap addressed and its principal scientific contribution. The mapping reflects the multidisciplinary scope of the Special Issue, encompassing hepatology, cardiology, immunology, genetics, diagnostics, epidemiology, pediatrics, pharmacology, and nutrition.

Contribution	Knowledge Gap Addressed	Principal Contribution
Morawska et al. [1]	Mechanism; epidemiology; therapy	Comprehensive mechanistic and quantitative review of MASLD–AF crosstalk; pooled meta-analyses; therapeutic synthesis (GLP-1 RA, SGLT-2 inhibitor, statin, resmetirom evidence).
Różycka-Kosmalska et al. [5]	Clinical phenotype; AF life-course	First systematic prospective analysis of clinically inferred MASLD across PAF, PeAF, PermAF; defines the cardio-renal phenotype of advanced AF.
Méndez-García et al. [7]	Mechanism (immunology–RAS)	First hepatic-level evidence that Th2 cytokines predict ACE/ACE2 expression in MASLD; identifies IL-13 as differentially expressed in patients with HA.
Nguyen and Vo [4]	Population-specific diagnostics	Non-invasive index performance (TyG-BMI, FLI AUROC 0.89) in a Southeast Asian cohort; gender-stratified cutoffs.
Mosca et al. [3]	Pediatric biomarkers	81% accuracy of Hcy for fibrosis in 182 children; identifies Hcy as a bipotential biomarker of cardiovascular risk and hepatic injury.
López-González et al. [8]	Modifiable determinants; lifestyle	Population-scale definition of high-risk profile in 16,708 Spanish workers; magnitude of age and physical activity effects on MAFLD risk scales.
Kwon et al. [6]	Body composition; adolescent MASLD	Inverse correlation between skeletal muscle–fat ratio and NAFLD severity in 122 adolescents; independent of BMI.
Coste et al. [2]	Genetics; immune mechanism	OR 5.25–10.57 for variant genotypes of IL-17 and TLR4 polymorphisms in Romanian MASLD; inflammatory-marker rationale.
Yılmaz et al. [9]	Natural preventive therapy; obesity mechanistic link	Significant decreases in body weight, leptin, asprosin, glucose, lipids, TBARS, and HOMA-IR and an increase in total testosterone in HFD rats administered white tea.

ACE—angiotensin-converting enzyme; AF—atrial fibrillation; AUROC—area under the receiver operating characteristic curve; BMI—body mass index; FLI—fatty liver index; GLP-1 RA—glucagon-like peptide -1 receptor agonist; HA—systemic arterial hypertension; Hcy—homocysteine; HFD—high-fat diet; HOMA-IR—homeostatic model assessment for insulin resistance; IL-13—interleukin 13; IL-17—interleukin 17; MASLD—metabolic dysfunction-associated steatotic liver disease; OR—odds ratio; PAF—paroxysmal atrial fibrillation; PeAF—persistent atrial fibrillation; PermAF—permanent atrial fibrillation; RAS—renin–angiotensin system; SGLT-2—sodium–glucose cotransporter 2; TBARS—thiobarbituric acid reactive substances; Th2—T helper type 2 cells; TLR4—toll-like receptor 4; TyG-BMI—triglyceride–glucose–body mass index.

## Abbreviations

The following abbreviations are used in this manuscript:

AASLD	American Association for the Study of Liver Diseases
ACE	Angiotensin-converting enzyme
AF	Atrial fibrillation
AIP	Atherogenic index of plasma
Ang 1-7	Angiotensin 1-7
ApoB	Apolipoprotein B
APRI	Aspartate aminotransferase to platelet ratio index
ASH	Alcoholic steatohepatitis
AUROC	Area under the receiver operating characteristic curve
BMI	Body mass index
CAC	Coronary artery calcium
CAD	Coronary artery disease
CI	Confidence interval
CKM	Cardiovascular–kidney–metabolic
CKD	Chronic kidney disease
CRHM	Cardiovascular–renal–hepatic–metabolic
CVD	Cardiovascular disease
EASL	European Association for the Study of the Liver
EGCG	Epigallocatechin-3-gallate
ELISA	Enzyme-linked immunosorbent assay
FDA	Food and Drug Administration
FGF-21	Fibroblast growth factor 21
FIB-4	Fibrosis-4 index
FLI	Fatty liver index
GLP-1 RA(s)	Glucagon-like peptide-1 receptor agonist(s)
HA	Systemic arterial hypertension
Hcy	Homocysteine
HDL-C	High-density lipoprotein cholesterol
HFD	High-fat diet
HF	Chronic heart failure
HFpEF	Heart failure with preserved ejection fraction
HFrEF	Heart failure with reduced ejection fraction
HOMA-IR	Homeostatic model assessment for insulin resistance
HR	Hazard ratio
IL-4	Interleukin-4
IL-10	Interleukin-10
IL-13	Interleukin-13
IL-17A	Interleukin-17A
IL-17F	Interleukin-17F
JAK1	Janus kinase 1
JAK2	Janus kinase 2
KCNN3	Potassium calcium-activated channel subfamily N member 3
LAP	Lipid accumulation product
LDL-C	Low-density lipoprotein cholesterol
MAFL	Metabolic dysfunction-associated fatty liver
MAFLD	Metabolic dysfunction-associated fatty liver disease
MASH	Metabolic dysfunction-associated steatohepatitis
MASLD	Metabolic dysfunction-associated steatotic liver disease
MetALD	MASLD + moderate alcohol intake
MFR	Muscle-to-fat ratio
MYH6	Myosin heavy chain 6
NAFLD	Non-alcoholic fatty liver disease
OR	Odds ratio
PAF	Paroxysmal atrial fibrillation
PCR-RFLP	Polymerase chain reaction-restriction fragment length polymorphism
PeAF	Persistent atrial fibrillation
PermAF	Permanent atrial fibrillation
PITX2	Paired-like homeodomain transcription factor 2
PNPLA3	Patatin-like phospholipase domain-containing protein 3
RAS	Renin–angiotensin system
ROC	Receiver operating characteristic
SGLT-2	Sodium–glucose cotransporter 2
STAT6	Signal transducer and activator of transcription 6
T2DM	Type 2 diabetes mellitus
TBARS	Thiobarbituric acid reactive substances
TG/HDL	Triglycerides-to-HDL ratio
Th2	T helper type 2 cells
TLR4	Toll-like receptor 4
TM6SF2	Transmembrane 6 superfamily member 2
TTN	Titin
TyG	Triglyceride–glucose index
TyG-BMI	Triglyceride–glucose–body mass index
TyG-WC	Triglyceride–glucose–waist circumference
